# A Novel Temperature Drift Error Estimation Model for Capacitive MEMS Gyros Using Thermal Stress Deformation Analysis

**DOI:** 10.3390/mi15030324

**Published:** 2024-02-26

**Authors:** Bing Qi, Jianhua Cheng, Zili Wang, Chao Jiang, Chun Jia

**Affiliations:** College of Intelligent Systems Science and Engineering, Harbin Engineering University, Harbin 150001, China; chengjianhua@hrbeu.edu.cn (J.C.); wangzili@hrbeu.edu.cn (Z.W.); jiangchaogcdx@hrbeu.edu.cn (C.J.); jiachun@hrbeu.edu.cn (C.J.)

**Keywords:** capacitive MEMS Gyros, temperature dependence, thermal stress deformation analysis, TDE precise test method based on heat flux analysis, RBFNN

## Abstract

Because the conventional Temperature Drift Error (TDE) estimation model for Capacitive MEMS Gyros (CMGs) has inadequate Temperature Correlated Quantities (TCQs) and inaccurate parameter identification to improve their bias stability, its novel model based on thermal stress deformation analysis is presented. Firstly, the TDE of the CMG is traced precisely by analyzing its structural deformation under thermal stress, and more key decisive TCQs are explored, including ambient temperature variation ∆*T* and its square ∆*T*^2^, as well its square root ∆*T*^1/2^; then, a novel TDE estimation model is established. Secondly, a Radial Basis Function Neural Network (RBFNN) is applied to identify its parameter accurately, which eliminates local optimums of the conventional model based on a Back-Propagation Neural Network (BPNN) to improve bias stability. By analyzing heat conduction between CMGs and the thermal chamber with heat flux analysis, proper temperature control intervals and reasonable temperature control periods are obtained to form a TDE precise test method to avoid time-consuming and expensive experiments. The novel model is implemented with an adequate TCQ and RBFNN, and the Mean Square Deviation (*MSD*) is introduced to evaluate its performance. Finally, the conventional model and novel model are compared with bias stability. Compared with the conventional model, the novel one improves CMG’s bias stability by 15% evenly. It estimates TDE more precisely to decouple Si-based materials’ temperature dependence effectively, and CMG’s environmental adaptability is enhanced to widen its application under complex conditions.

## 1. Introduction

Capacitive MEMS Gyros (CMGs) have so many merits, such as significant miniaturization, high integration, low power consumption, and cost, and they are widely utilized and applied in diverse fields, for example, inertial navigation, attitude detection, and heading guidance [1,2,3,4]. However, because CMGs are manufactured with Si-based materials of temperature dependence, temperature must change the physical properties of Si-based materials, which introduces Temperature Drift Error (TDE) to reduce CMG’s bias stability and limits their applications in some fields [5]. Intrinsically, optimizing the production process of Si-based materials to decouple their temperature dependence is the fundamental way to eliminate TDE [6]. Due to current material process limitations, it is so hard to decouple temperature dependence in the present technological context. In the background of the great repeatability of CMGs, temperature control and mathematical estimation are mainstream methodologies to eliminate TDE [7,8,9]. The first one uses a temperature control system to stabilize ambient temperature, and it has the merits of high temperature control accuracy. In contrast, its demerits, high power consumption, large volume, and complex control, limit it to be used on a large scale [10,11,12]. The second one is based on given models to estimate TDE precisely and calibrate CMG’s outputs in real time. Although it has the merits of low cost and easy implementation, its accuracy depends on precise TDE traceability and parameter identification of TDE estimation models [13,14].

On one hand, TDE traceability is important to enhance CMG’s bias stability [15]. Considering the consistent working principle of capacitive MEMS devices, Temperature Correlated Quantities (TCQs), exciting TDE is universal and invariant, and it ought to be figured out completely to offer references to describe TDE more accurately. Based on many experiments, Maj et al. show ambient temperature variations of 1 °C introduce a sensitivity variation of 1% of the scale factor of a capacitive MEMS accelerometer, which indicates that the direct cause of TDE is ambient temperature [16]. Kim et al. research the influence of ambient temperature on three-dimensional sizes of Si-based materials, and the experimental results point out that Si-based materials deform linearly with ambient temperature [17]. So, a composite model for estimating TDE is built with ambient temperature and TDE. After estimation and compensation, the bias stability of capacitive MEMS devices is improved by 10%. Wen et al. figured out that ambient temperature variation is key to describing structural deformation inside CMGs [18]. Ambient temperature and its variation are utilized as the TCQ of TDE, and the Radial Basis Function Neural Network (RBFNN) is used to estimate TDE precisely; then, CMG’s bias stability is improved by 10%. Bekkeng explores TCQs, ambient temperature, and their variation’s square, and a Kalman filter is applied to estimate TDE [19]. Then, the absolute rate error of MEMS inertial devices is reduced by more than a factor of 10. Chen et al. use linear microstructure thermal analysis to show microstructure deformation of the combs in MEMS capacitive accelerometers and obtain TCQs, ambient temperature variation, and their variation’s square [20]. TDE is estimated with TCQs and RBFNNs, and its bias stability is improved by 60%. But, microstructure deformation is analyzed from an uncomprehensive perspective, and the factors that may cause nonlinear deformation are not studied to confirm whether nonlinear deformation occurs. Shi et al. propose a multi-physical analysis to research TDE [21]. It reveals that the deviation of the thermal expansion coefficient between simulations and experiments is about 1~2 mg/°C, and thermal expansion properties of Si-based materials are nonlinear, which is non-ignorable to TDE traceability. Ambient temperature and its square and ambient temperature variation and its square are explored as TCQs, and a novel TDE estimation model is established with them and a Back Propagation Neural Network (BPNN) using particle swarm optimization plus genetic algorithm. The experimental results show that its bias stability is improved by 16.01% compared with the conventional model. Furthermore, a conclusion is formed that Si-based materials have complex structural deformation under the action of bending moment and elastic modulus, and it further shows that partial structural deformation inside CMGs is nonlinear [22,23,24,25]. So, the key to tracing TDE accurately lies in exploring complete TCQs based on the nonlinear structural deformation of Si-based materials, especially under thermal stress.

On the other hand, precise parameter identification which describes the relationship between TCQs and TDE is another key factor, and it requires the prerequisites that the TDE estimation model is accurate. An accurate TDE estimation model focuses on the relationship between its inputs and outputs, and the bias stability of CMGs is compensated to the target ones with its inputs [26]. Cheng et al. apply a particle swarm optimization algorithm in optimizing support vector machine models to improve bias stability. Small batch data processing methods are used to ensure its modeling in real time [27]. Although it improves capacitive MEMS accelerometers’ bias stability by up to 18.96%, support vector machine models with complicated structures are unsuitable for disposing a lot of experimental results and even reduce in real time. Pan et al. show a TDE estimation model using a wavelet neural network, which improves bias stability after compensation to 10% of that before [28]. Due to the diversity and uncertainty of signal transmission in wavelet neural networks, extensive training is needed to identify its precise parameters. Xu et al. propose a TDE estimation model based on a BPNN, and the nonlinear maximum of TDE is reduced from 3329 ppm to 603 ppm [29]. As a kind of classical artificial neural network, the BPNN has a simple structure, including an input layer, one or more hidden layers, and an output layer, which achieve more outstanding estimation performance to ensure its higher precision and better real time performance, as well as easier implementation in applications compared with other methods [30]. As is known, when BPNNs are trained, their local optimums are prone to occur and may introduce non-optimal estimates of TDE. Wang et al. use a genetic algorithm to assist in training BPNNs to avoid them [31]. The bias stability of the compensated capacitive MEMS accelerometer is less than 0.017% during −10~80 °C, and it is 173 times more precise than the TDE estimation model with polynomial fitting. But, the genetic algorithm has the demerits of probability disorder, reducing its calculation accuracy and real time performance and increasing the training amount of BPNNs and the targeted model’s identification difficulty. So, a proper parameter identification is able to describe the complex nonlinearity and estimate TDE precisely. Additionally, it should have more real time to compensate for CMG’s bias stability with its TCQs to ensure that it outputs angular velocity timely, even in the case that the carrier rotates at a large angular velocity. Compared with the BPNN, the RBFNN is a neural network based on the function approximation method with three neural layers. The function approximation method ensures that it describes the targeted complex nonlinearity as accurately as possible, and three neural layers reduce calculation time to guarantee TDE estimated in real time [32]. So, the RBFNN is a more proper choice compared to other neural networks. However, inaccurate experimental results result in its imprecise implementation to influence its estimation accuracy, so accurate experimental results are an important premise to ensure RBFNN’s performance.

In the paper, TDE traceability for CMGs is explored using thermal stress deformation analysis by simulating structural deformation under diverse conditions, and all-new TCQs are obtained as the key factors to TDE traceability, ambient temperature variation ∆*T,* and its square ∆*T*^2^, as well as its square root ∆*T*^1/2^. To improve TDE estimation performance, an RBFNN is used to modify the conventional model based on a BPNN and establish a novel model. Moreover, by analyzing the heat conduction between CMGs and thermal chambers principally, a precise TDE test is established, and two important characteristics are determined: proper temperature control interval and a reasonable temperature control period. The novel model estimates TDE more accurately to decouple the temperature dependence of Si-based materials, which further enhances the environmental adaptability of CMGs.

## 2. Methodology

### 2.1. Precise TDE Traceability

#### 2.1.1. Conventional TDE Estimation Model

**Figure 1:**
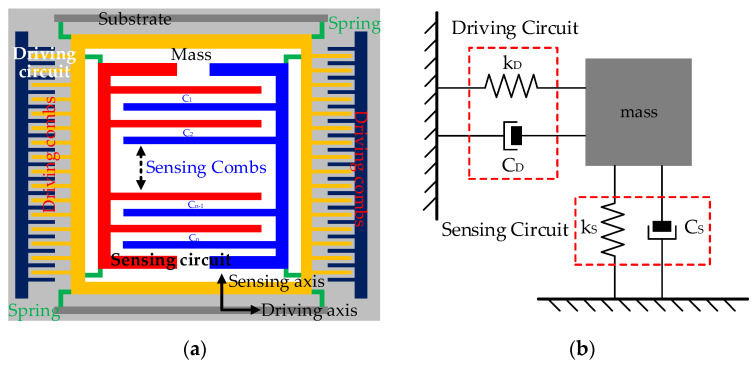
Basic principle of the CMG. (**a**) Physical design; (**b**) working principle.

CMGs are miniaturized devices manufactured with Si-based materials and include mass, driving circuit, sensing circuit, and substrate. With microelectromechanical technology, they are assembled as microelectromechanical units. Figure 1 shows their basic principles.

Where *k_D_* and *k_S_* are the springs of the driving circuit and sensing circuit, respectively, and *C_D_* and *C_S_* are their corresponding capacitors. According to Figure 1, the sensing circuit and driving circuit have interspersed combs, and driving combs and sensing combs are movable. Under the action of driving combs, sensing combs move along the driving axis. Because of air gaps between driving combs, they can be abstracted as plate capacitors. One capacitance of sensing combs *C*_0_ is shown with the plate capacitor’s formula as follows:

(1) $$C_{0}=\frac{\varepsilon }{4\pi k}\frac{S_{0}}{d_{0}}$$

where *ε* is the relative dielectric constant, *k* is the electrostatic force constant, *S*_0_ is the overlap area between the sensing combs, and *d*_0_ is its plate distance. In Figure 1, when the carriers rotate, sensing combs displace away from their original position under the action of the Coriolis force, and their displacements are related to the carriers’ angular velocity. The higher their angular velocity is, the greater air gaps between the sensing combs change, and the more significantly their capacitance varies. Because sensing combs have a common end, they are connected in parallel in terms of electrical form. Assuming that sensing combs have 2n combs, there are *n* air gaps, and capacitance variation ∆*C* measured by CMGs is shown as follows:

(2) $$\Delta C=\sum_{i=1}^{n}\left(C_{i}-C_{0}\right)=n\left[\frac{\varepsilon }{4\pi k}\frac{S_{0}}{\left(d_{0}-\Delta d\right)}-\frac{\varepsilon }{4\pi k}\frac{S_{0}}{d_{0}}\right]$$

where ∆*d* is plate distance variation and *C_i_* is the capacitance between each air gap. So, the carriers’ rotation is precisely sensed with capacitance variation. Also, sensing and driving circuits’ stiffness determines CMG’s structural consistency. Considering that their stiffness is so related to Si-based materials’ stiffness, ambient temperature is considered the root cause [18]. Sensing and driving circuits deform as the ambient temperature varies inevitably and the capacitance errors of sensing combs are excited, as are the TDE ∆*E* in CMG’s output. The conventional TDE estimation model describes CMGs’ structural deformation using linear analytical methods and shows that ∆*E* is determined by ambient temperature variation ∆*T* and its square ∆*T*^2^. So, the conventional model is shown as follows:

(3) $$\Delta E_{cap}\propto \left(\Delta T,\Delta T^{2}\right)$$

#### 2.1.2. A Novel TDE Estimation Model with Thermal Stress Deformation Analysis

According to Figure 1, the mass, driving circuit, and sensing circuit are fabricated on the substrate, and they displace together as a whole with the substrate. Even if it deforms as ambient temperature, their relative distances between each other remain relatively stable, especially the air gaps. So, the substrate’s structural deformation has no influence on CMG’s performance, and it can be negligible. In contrast, the structural deformation of sensing combs changes air gaps between them and worsens the accuracy of capacitance variation. So, their structural deformation is the key factor to be considered comprehensively.

Thermal stress is an internal stress inside objects. When ambient temperature varies, the stress generated by the objects due to external and mutual constraints makes its structure unable to fully expand and contract freely. It makes CMG’s structure deform under the excitation of ambient temperature variation and changes the consistency of air gaps between the sensing combs, and the accuracy of capacitance measurements are reduced directly. Then, the influence of thermal stress on the capacitance measurement of sensing combs is analyzed using thermal stress deformation analysis in diverse conditions.

(a) Ambient temperature *T* = *T*_0_ and angular velocity *ω* = *ω*_0_

**Figure 2:**
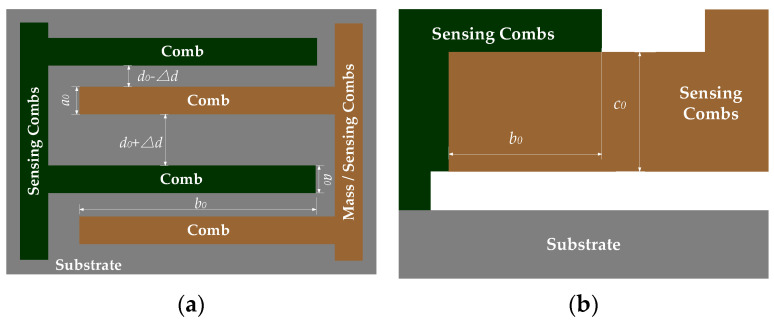
Partial view of the structure of sensing combs when *T* = *T*_0_ and *ω* = *ω*_0_. (**a**) Top view of sensing combs; (**b**) side view of sensing combs.

When ambient temperature stabilizes at *T*_0_, CMG’s structure stays stable. If the carriers rotate at *ω* = *ω*_0_, sensing combs displace in the *y*-axis under the Coriolis force. Figure 2 shows the structure of sensing combs when *T* = *T*_0_ and *ω* = *ω*_0_.

Where *a*_0_ is the comb thickness of sensing combs, *b*_0_ is the length of overlap area between the sensing combs, *c*_0_ is their width, and *S*_0_ = *b*_0_*c*_0_. Because ambient temperature stays still at *T*_0_, CMG’s structure remains unchanging, and the capacitance of the sensing combs is constant. According to (2), ∆*C* measured by CMGs is shown as follows:

(4) $$\Delta C=\sum_{i=1}^{n}\left(C_{i}-C_{0}\right)=\frac{n\varepsilon }{4\pi k}\left(\frac{b_{0}c_{0}}{d_{0}-\Delta d}-\frac{b_{0}c_{0}}{d_{0}}\right)$$

(b) Ambient temperature *T* = *T*_1_ and angular velocity *ω* = *ω*_0_

**Figure 3:**
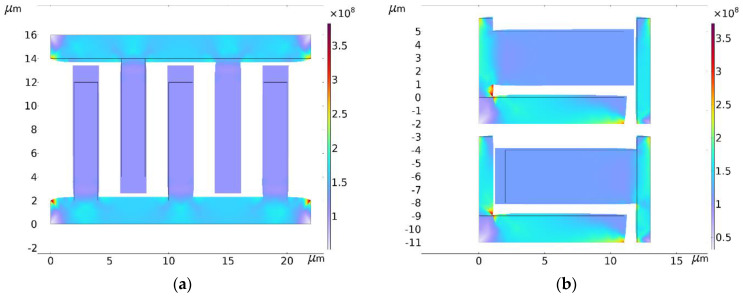
Partial view of the structure of sensing combs under thermal stress with COMSOL Multiphysics 6.0 when *T* = *T*_1_ and *ω* = *ω*_0_. (**a**) Top view in simulations; (**b**) side view in simulations.

When *T* = *T*_0_**,** it is unmistakable for sensing combs to deform in three dimensions because of the temperature dependence of Si-based materials. According to Thermal Expansion Theory, structural deformation includes expanding or contracting, which changes the air gaps of sensing combs and their capacitance. So, the multi-physical field simulation software COMSOL Multiphysics 6.0 is used to show its structural deformation. Because CMG’s structure is at a micrometer scale, about 1~100 μm, specific physical quantities of CMG’s structure are set at a common value [33,34]. The thermal expansion coefficient of Si-based materials is 2.4 × 10^−6^/°C, its elastic modulus is 175 GPa, and its Poisson’s ratio is 0.28. Figure 3 shows the structure of sensing combs under thermal stress when *T* = *T*_1_ and *ω* = *ω*_0_ in simulations with COMSOL Multiphysics 6.0.

According to Figure 3, the combs have complex structural deformations under thermal stress, including linear and nonlinear. For non-connecting ends of the sensing combs, which have structural characteristics of long beams of Si-based materials, they deform freely in three dimensions following the linear thermal expansion formula. After deforming, its structure is still a long beam whose three-dimensional sizes scale up or down proportionally as the ambient temperature varies [35]. In contrast, thermal stress from the connecting ends acts on sensing combs, limiting their free structural deformation, and the bending moment from thermal stress brings local nonlinear deformation. According to (4), the capacitance variation of sensing combs is related to the overlap area of the sensing combs and their plate distance. According to Figure 3, some characteristics of structural deformation are obtained as follows:

- Thermal stress from the connecting ends limits sensing combs to deform laterally, like its width and thickness, and it is useless for longitudinal structural deformation at all. So, its length deforms freely and can be calculated with a linear thermal expansion formula.
- Thermal stress from the connecting ends and free structural deformation of the non-connecting ends make sensing combs’ length vary in a curved line. Given that its width is bigger than its thickness in normal conditions, thermal stress to the width is also bigger than that to its thickness, which means the curve radian of the thickness varies more quickly than the width. Considering that sensing combs are arranged in a positive and negative way, the overlap area is represented as an approixmate rectangle whose length and width should be calculated with the linear thermal expansion formula.
- According to Figure 3, because the the curve radian of the thickness of the overlap area varies much more quickly than its width, the distance between the sensing combs is shown as a “wide-narrow-wide” pattern. It causes plate distance to vary nonlinearly. In a word, it should be calculated with a nonlinear expression.

**Figure 4:**
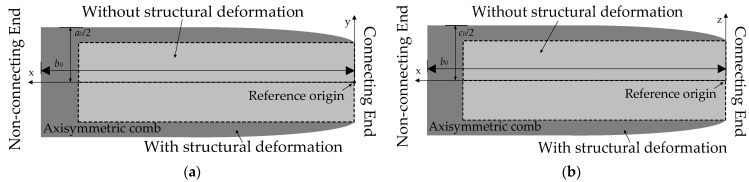
Structural deformation of long beams with and without thermal stress. (**a**) Top view of the long beam; (**b**) side view of the long beam.

Based on that, the length of the overlap area *b*_1_ with linear thermal expansion formula can be shown as follows:

(5) $$b_{1}=b_{0}+\Delta b^{\prime }+\Delta b^{\prime \prime }=b_{0}+\alpha b_{0}\Delta T+\alpha b_{0}\Delta T=b_{0}\left(1+2\alpha \Delta T\right)$$

where *α* is the thermal expansion coefficient of Si-based materials, ∆*b*′ is the length deformation of a sensing comb, ∆*b*″ is that of the next one, and ∆*T* is ambient temperature variation. Then, the width of the overlap area with linear thermal expansion formula *c*_1_ is shown as follows:

(6) $$c_{1}=c_{0}+2\Delta c=c_{0}+2\alpha c_{0}\Delta T=c_{0}\left(1+2\alpha \Delta T\right)$$

where ∆*c* is width deformation. According to Figure 3, the structural deformation of sensing combs is near the connecting end, which influences the in-plate distance of the overlap area but not its area. In a word, the area of the overlap area can be seen as constant, but its plate distance varies at any point of sensing combs. Figure 4 shows the structural deformation of the long beam of Si-based materials under thermal stress.

Given that the sensing combs are axisymmetric, a three-dimensional positive half-axis coordinate system is established on a comb for simplified calculation in Figure 4. Additionally, the bending moment from thermal stress is used to describe nonlinear structural deformation. So, its bending moment *M* at any position of the sensing combs can be calculated as follows [36]:

(7) $$M=\int_{0}^{\frac{a_{0}}{2}}\alpha E\Delta Tb_{0}\left(\frac{a_{0}}{2}-x\right)dx=\frac{\alpha Eb_{0}a_{0}^{2}}{8}\Delta T$$

where *E* is the elastic modulus of Si-based materials. According to Figure 4, the thickness of sensing combs *a*_1_ under its bending moment is shown as follows:

(8) $$\begin{array}{cc} a_{1}=a_{0}+2\Delta a=a_{0}+2\frac{M}{EI}\frac{x^{2}}{2}=a_{0}+12\frac{\alpha \Delta T}{a_{0}}x^{2} & x\in \left(0,b_{0}\right) \end{array}$$

where ∆*a* is thickness deformation, *I* is the inertia moment of the combs, and *I = b*_0_(*a*_0_/2)^3^/12. So, the plate distance under its bending moment *d*_1_ is shown as follows:

(9) $$d_{1}=d_{0}-\Delta a_{P}-\Delta a_{N}=d_{0}-12\frac{\alpha \Delta T}{a_{0}}x^{2}-\left[12\frac{\alpha \Delta T}{a_{0}}{\left(b_{0}-x\right)}^{2}\right]=d_{0}-12\frac{\alpha \Delta T}{a_{0}}\left(2x^{2}-2b_{0}x+b_{0}^{2}\right)$$

where ∆*a_P_* is the thickness deformation of sensing combs arranged in a positive way and ∆*a_N_* is the thickness deformation arranged in a negative way. If the carriers rotate at *ω* = *ω*_0_, the capacitance in the sensing circuit ∆*C*′ is shown as follows:

(10) $$\begin{array}{cc} \Delta C^{\prime } & =\sum_{i=1}^{n}\left({C^{\prime }}_{i}-{C^{\prime }}_{0}\right)=n\int_{0}^{b_{0}}\left(\frac{\varepsilon }{4\pi k}\frac{b_{1}c_{1}}{d_{1}-\Delta d}-\frac{\varepsilon }{4\pi k}\frac{b_{1}c_{1}}{d_{1}}\right)dx\\ & =\frac{n\varepsilon }{4\pi k}b_{0}c_{0}\left(1+2\alpha \Delta T\right)\left(1+2\alpha \Delta T\right)\int_{0}^{b_{0}}\left[\frac{1}{d_{0}-12\frac{\alpha \Delta T}{a_{0}}\left(2x^{2}-2b_{0}x+b_{0}^{2}\right)-\Delta d}-\frac{1}{d_{0}-12\frac{\alpha \Delta T}{a_{0}}\left(2x^{2}-2b_{0}x+b_{0}^{2}\right)}\right]dx\\ & =\frac{n\varepsilon b_{0}c_{0}}{4\pi k}{\left(1+2\alpha \Delta T\right)}^{2}\frac{1}{24\frac{\alpha \Delta T}{a_{0}}}\int_{0}^{b_{0}}\left[\frac{1}{-{\left(x-\frac{b_{0}}{2}\right)}^{2}+\frac{d_{0}-\Delta d}{24\frac{\alpha \Delta T}{a_{0}}}-\frac{b_{0}^{2}}{4}}-\frac{1}{-{\left(x-\frac{b_{0}}{2}\right)}^{2}+\frac{d_{0}}{24\frac{\alpha \Delta T}{a_{0}}}-\frac{b_{0}^{2}}{4}}\right]dx\\ & =\frac{n\varepsilon b_{0}c_{0}}{4\pi k}{\left(1+2\alpha \Delta T\right)}^{2}\frac{1}{4\sqrt{6\frac{\alpha \Delta T}{a_{0}}}}\left(\frac{1}{\sqrt{d_{0}-\Delta d-24\frac{\alpha \Delta T}{a_{0}}\frac{b_{0}^{2}}{4}}}\ln\left|\frac{\frac{b_{0}}{2}+\sqrt{\frac{d_{0}-\Delta d}{24\frac{\alpha \Delta T}{a_{0}}}-\frac{b_{0}^{2}}{4}}}{-\frac{b_{0}}{2}+\sqrt{\frac{d_{0}-\Delta d}{24\frac{\alpha \Delta T}{a_{0}}}-\frac{b_{0}^{2}}{4}}}\right|-\frac{1}{\sqrt{d_{0}-24\frac{\alpha \Delta T}{a_{0}}\frac{b_{0}^{2}}{4}}}\ln\left|\frac{\frac{b_{0}}{2}+\sqrt{\frac{d_{0}}{24\frac{\alpha \Delta T}{a_{0}}}-\frac{b_{0}^{2}}{4}}}{-\frac{b_{0}}{2}+\sqrt{\frac{d_{0}}{24\frac{\alpha \Delta T}{a_{0}}}-\frac{b_{0}^{2}}{4}}}\right|\right) \end{array}$$

In (2), the capacitance errors measured in sensing circuit ∆*E_cap_*′ can be expressed as follows:

(11) $$\begin{array}{cc} \Delta E_{cap}\prime & =\Delta C^{\prime }-\Delta C\\ & =\frac{n\varepsilon b_{0}c_{0}}{4\pi k}{\left(1+2\alpha \Delta T\right)}^{2}\frac{1}{4\sqrt{6\frac{\alpha \Delta T}{a_{0}}}}\left(\frac{1}{\sqrt{d_{0}-\Delta d-24\frac{\alpha \Delta T}{a_{0}}\frac{b_{0}^{2}}{4}}}\ln\left|\frac{\frac{b_{0}}{2}+\sqrt{\frac{d_{0}-\Delta d}{24\frac{\alpha \Delta T}{a_{0}}}-\frac{b_{0}^{2}}{4}}}{-\frac{b_{0}}{2}+\sqrt{\frac{d_{0}-\Delta d}{24\frac{\alpha \Delta T}{a_{0}}}-\frac{b_{0}^{2}}{4}}}\right|-\frac{1}{\sqrt{d_{0}-24\frac{\alpha \Delta T}{a_{0}}\frac{b_{0}^{2}}{4}}}\ln\left|\frac{\frac{b_{0}}{2}+\sqrt{\frac{d_{0}}{24\frac{\alpha \Delta T}{a_{0}}}-\frac{b_{0}^{2}}{4}}}{-\frac{b_{0}}{2}+\sqrt{\frac{d_{0}}{24\frac{\alpha \Delta T}{a_{0}}}-\frac{b_{0}^{2}}{4}}}\right|\right)-\frac{n\varepsilon }{4\pi k}\left(\frac{b_{0}c_{0}}{d_{0}-\Delta d}-\frac{b_{0}c_{0}}{d_{0}}\right)\\ & =f_{1}\left(\Delta T,\Delta T^{2}\right)f_{2}\left(\sqrt{\Delta T}\right)f_{3}\left(\sqrt{\Delta T}\right)\propto f\left(\Delta T,\Delta T^{2},\sqrt{\Delta T}\right) \end{array}$$

According to (11), there is a deviation between CMG’s output and its reference when ambient temperature varies. (11) can be expressed as the product of three uncorrelated functions that cannot be simplified further, and it is related to ambient temperature variation ∆*T* and its square ∆*T*^2^, as well as its square root ∆*T*^1/2^. So, the conventional model is modified to a novel model, which is shown as follows:

(12) $$\Delta E_{cap}\prime \propto f\left(\Delta T,\Delta T^{2},\Delta T^{1/2}\right)$$

### 2.2. Precise Parameter Identification for the Novel TDE Precise Estimation Model

Based on precise TDE traceability and a novel model for CMGs, identifying its parameters is another important factor. The more precisely the novel model is identified, the less ambient temperature influences TDE, and the better the bias stability of CMGs. So, testing TDE accurately is the key prerequisite, and a proper TDE test method is essential.

#### 2.2.1. TDE Accurate Acquisition Methodology

(a) Heat conduction solutions

To ensure the experimental results’ reliability, heat from ambient temperature should be conducted to CMGs efficiently. Heat conduction solutions are applied on CMGs, like thermal grease and thermal conductive rubber, which keep the ambient temperature the same as the CMG and reduce the deviation between experimental results and their theoretical values.

(b) Precise temperature measurement system

Precise ambient temperature represents CMG’s environmental adaptability comprehensively, so it is necessary to apply a precise temperature measurement system. It needs be installed closely on the CMG to reduce the temperature gradient. Its measuring accuracy needs to be over 2 times more accurate than ambient temperature variation, and its measuring frequency needs to be higher than CMG’s output frequency for complete experimental results.

(c) Proper temperature control interval

TDE is a statistical characteristic deviation represented based on mathematical models. Once CMGs are manufactured, their mechanical properties are fixed, and their TDE can be expressed with bias, scale factor, and random error. So, CMGs have similar environmental adaptability but unique TDEs, and from their datasheets, TDEs can be roughly estimated as follows:

(13) $$\Delta E^{\prime }=\gamma \Delta T+\beta \Delta T$$

where ∆*E*′ are roughly estimated TDEs, *γ* is the parameter “Zero-rate level change vs. temperature”, and *β* is the parameter “Sensitivity change vs. temperature”. Theoretically, ∆*E*′ is smaller than TDE because some non-statistical errors are ignored and ∆*E*′ ≤ ∆*E*. CMG’s sensitivity ∆*E_S_* determines the measured minimum of angular velocity, and higher sensitivity brings more accurate angular velocity. When ambient temperature deteriorates suddenly during CMG work, it is probable that TDE is much bigger than ∆*E_S_*, even covering up CMG’s sensitivity to measure inaccurate angular velocity. So, it is essential to vary ambient temperature at a reasonable temperature variation rate. To test TDE precisely, set ∆*E_S_* ≈ ∆*E*′, and the temperature control interval can be further expressed as follows:

(14) $$\Delta T\leq \frac{\Delta E_{S}}{\gamma +\beta }$$

(d) Reasonable temperature control period

**Figure 5:**
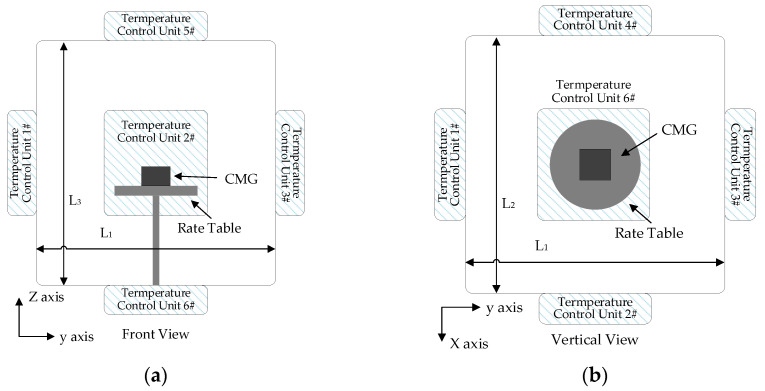

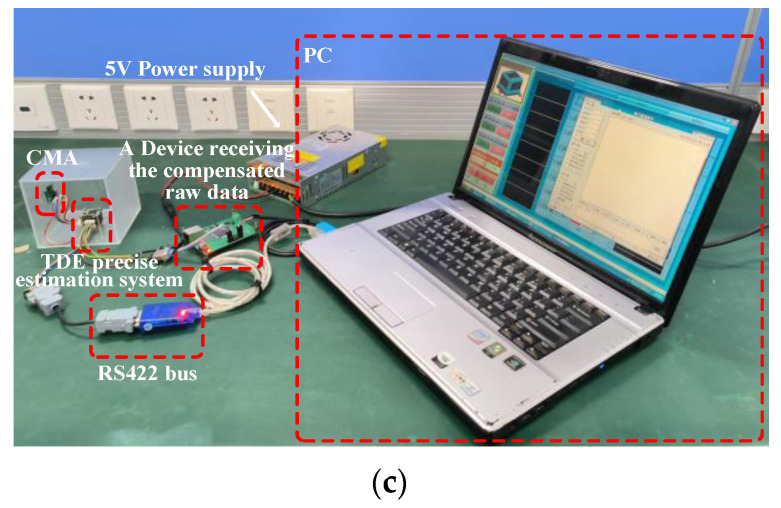
A schematic diagram of the test platform. (**a**) Front view of the CMG installation; (**b**) vertical view of the CMG installation; (**c**) field test of the CMG.

According to heat conduction theory, a long enough heat conduction period conducts the heat from position A to position B completely, which ensures that ambient temperature variation at position A is the same as position B. An insufficient heat conduction period causes an incomplete heat conduction process, and the TDE of the CMG is tested imprecisely. Usually, a thermal chamber is used to test TDE. It is designed as a cube of front-door-open and -closed insulation. Also, it integrates a temperature control system with six temperature control units on each plane, and they can be further simplified according to the targeted control efficiency. There is a rate table inside to offer the referenced angular velocity. To reflect the ambient temperature of the CMG better, temperature sensors are set on it. Figure 5 shows a schematic diagram of the test platform.

In Figure 5, the heat is transferred through the thermal chamber’s inner wall to control ambient temperature inside the targets, and the heat from temperature control units uniformly transfers to the CMG. Then, the center of the rate table is taken as a reference position, and it is also the central position of the thermal chamber. Given that the sizes of the thermal chamber are *L*_1_ *× L*_2_ *× L*_3_, the farther the position is away from the inner wall, the longer the period of heat transfers. Under the action of temperature control units, CMGs obtain the heat, and their environmental adaptability changes. The position where the CMG is located is the final area where the ambient temperature stays stable. According to energy conservation law and Newton’s law of cooling, the heat flux density equation in three dimensions is shown as follows:

(15) $$q=-k_{h}\left(\frac{\partial u}{\partial x}\vec{i}+\frac{\partial u}{\partial y}\vec{j}+\frac{\partial u}{\partial z}\vec{k}\right)$$

where *u* is the ambient temperature of any position in the thermal chamber at moment *t*, *q* is its heat flux density, *k_h_* is the heat conductivity coefficient, ∂u/∂x is the spatial varying rate of ambient temperature on the *x*-axis, ∂u/∂y is the spatial varying rate of the ambient temperature on the *y*-axis, ∂u/∂z is the spatial varying rate of the ambient temperature on the *z*-axis, $\vec{i}$ is the unit vector on the *x*-axis, $\vec{j}$ is the unit vector on the *y*-axis, and $\vec{k}$ is the unit vector on the *z*-axis. The heat on the *x*-axis heats CMGs, and according to (15), a heat transfer equation can be established as follows:

(16) $$Q_{x}=\left[{\left(q_{x}\right)}_{A}-{\left(q_{x}\right)}_{A+\Delta A}\right]\Delta y\Delta z\Delta t=\left[{\left(-k_{h}\frac{\partial u}{\partial x}\right)}_{A}-{\left(-k_{h}\frac{\partial u}{\partial x}\right)}_{A+\Delta A}\right]\Delta y\Delta z\Delta t=-k_{h}\frac{\partial^{2}u}{\partial x^{2}}\Delta x\Delta y\Delta z\Delta t$$

where *Q_x_* is the heat at position *A* on the *x*-axis, (*q_x_*)*_A_* is its heat flux density, (*q_x_*)*_A+_*_Δ_*_A_* is its heat flux density of its close position *A* + Δ*A* near position *A* on the *x*-axis, Δ*y* is the width of the plane where the heat conducts, Δ*z* is the height of the plane where the heat conducts, and Δ*t* is the period when the heat conducts. Considering that the CMG is heated on the *x*-axis, *y*-axis, and *z*-axis, a heat transfer equation can be established on the *y*-axis and *z*-axis as follows:

(17) $$\begin{array}{cc} Q_{y}=-k_{h}\frac{\partial^{2}u}{\partial y^{2}}\Delta x\Delta y\Delta z\Delta t & Q_{z}=-k_{h}\frac{\partial^{2}u}{\partial z^{2}}\Delta x\Delta y\Delta z\Delta t \end{array}$$

where *Q_y_* is the heat at position *A* on the *y*-axis and *Q_z_* is the heat of position *A* on the *z*-axis. Based on (16) and (17), the heat at position *A* in three dimensions can be deduced as follows:

(18) $$Q=Q_{x}+Q_{y}+Q_{z}=-k_{h}\Delta x\Delta y\Delta z\Delta t\left(\frac{\partial^{2}u}{\partial x^{2}}\vec{i}+\frac{\partial^{2}u}{\partial y^{2}}\vec{j}+\frac{\partial^{2}u}{\partial z^{2}}\vec{k}\right)+F\left(x,y,z,t\right)\Delta x\Delta y\Delta z\Delta t\vec{v}$$

where *F*(*x*,*y*,*z*,*t*) is the heat flux density of the potential heat sources related to its position and time, *v* is the 3D unit vector matrix, and $\vec{v}={\left[\begin{array}{ccc} \vec{i} & \vec{j} & \vec{k} \end{array}\right]}^{T}$. When the thermal chamber works in a period *t_s_*, according to the specific heat capacity formula, we can obtain the following equation:

(19) $$Q=\left[-k_{h}\Delta x\Delta y\Delta z\left(\frac{\partial^{2}u}{\partial x^{2}}\vec{i}+\frac{\partial^{2}u}{\partial y^{2}}\vec{j}+\frac{\partial^{2}u}{\partial z^{2}}\vec{k}\right)+F\left(x,y,z,t\right)\Delta x\Delta y\Delta z\vec{v}\right]t_{s}=Cm\Delta T\vec{v}$$

where *C* is the specific heat capacity of air in a thermal chamber in a closed condition, *m* is its mass, ∆*T* is temperature variation, and ∆*T =* |*T_b_-T*_0_|. *T*_0_ is the initial temperature and *T_b_* is the final temperature. Then, (19) can be transformed as follows:

(20) $$\left[-k_{h}\Delta x\Delta y\Delta z\left(\frac{\partial^{2}u}{\partial x^{2}}\vec{i}+\frac{\partial^{2}u}{\partial y^{2}}\vec{j}+\frac{\partial^{2}u}{\partial z^{2}}\vec{k}\right)+F\left(x,y,z,t\right)\Delta x\Delta y\Delta z\vec{v}\right]t_{s}=C\rho \Delta x\Delta y\Delta z\Delta T\vec{v}$$

where *ρ* is the air density inside the thermal chamber. Considering that different positions in the heat conduction pathway have different heat flux densities in the heat conduction process, the further the heat is conducted, the smaller the heat flux density becomes, and the lower heat transfer efficiency stays. The CMG is a sensor, and its heat is relatively insignificant to the thermal chamber, so *F*(*x*,*y*,*z*,*t*) can be ignored in magnitude. Then, (20) can be deduced as follows:

(21) $$\left(\frac{\partial^{2}u}{\partial x^{2}}\vec{i}+\frac{\partial^{2}u}{\partial y^{2}}\vec{j}+\frac{\partial^{2}u}{\partial z^{2}}\vec{k}\right)=-\frac{C\rho \Delta T}{k_{h}t_{s}}\vec{v}$$

To calculate total heat along the heat conduction pathway, (21) is integrated on the *x*-axis, *y*-axis, and *z*-axis separately, and then we obtain the following equation:

(22) $$\int_{0}^{L_{1}}\int_{0}^{L_{1}}\frac{\partial^{2}u}{\partial x^{2}}dxdx+\int_{0}^{L_{2}}\int_{0}^{L_{2}}\frac{\partial^{2}u}{\partial y^{2}}dydy+\int_{0}^{L_{3}}\int_{0}^{L_{3}}\frac{\partial^{2}u}{\partial z^{2}}dzdz=\Delta T\frac{C\rho }{k_{h}t_{s}}\left(\int_{0}^{L_{2}}\int_{0}^{L_{3}}dydz+\int_{0}^{L_{1}}\int_{0}^{L_{3}}dxdz+\int_{0}^{L_{1}}\int_{0}^{L_{2}}dxdy\right)$$

After integration, (22) is transformed and shown as follows:

(23) $$T_{x}+T_{y}+T_{z}=\Delta T\frac{C\rho }{k_{h}t_{s}}\left(L_{2}L_{3}+L_{1}L_{3}+L_{1}L_{2}\right)$$

where *Tx* is the control target of the temperature control unit on the *x*-axis, *Ty* is on the *y*-axis, and *Tz* is on the *z*-axis. Then, *t_s_* can be deduced as follows:

(24) $$t_{s}=\frac{C\rho }{k_{h}}\frac{\left(L_{2}L_{3}+L_{1}L_{3}+L_{1}L_{2}\right)}{\left(T_{x}+T_{y}+T_{z}\right)}\Delta T$$

Usually, a thermal chamber is designed as a cube, and its size is *L × L × L*. Moreover, the control target on the *x*-axis, *y*-axis, and *z*-axis is set the same as *T_b_*, and (24) can be simplified further as follows:

(25) $$t_{s}=\frac{C\rho L^{2}}{k_{h}T_{b}}\left(T_{b}-T_{0}\right)$$

Then, the period for heat conduction from the inner wall to the center of the thermal chamber can be calculated with (25). To guarantee that the thermal chamber is heated uniformly, the temperature control period is set as *t_s_* ≤ *t_p_.* Taking L3GD20H as an example, it is chosen to test TDE randomly. From its datasheet, ∆*E_S_* = 8.75 mdps/digit, *γ* = ±0.04 dps/°C, and its operating temperature range is −40~85 °C. After dimensional transformation, *β* is obtained as follows:

(26) $$\beta =2\%/{}^{\circ}C\times 8.75\mathrm{mdps}\times \frac{\left[\left(85\;{}^{\circ}C\right)-\left(-40\;{}^{\circ}C\right)\right]}{2}=10.9375\mathrm{mdps}/{}^{\circ}C$$

According to (14), temperature control interval ∆*T* is shown as follows:

(27) $$\Delta T\leq \frac{8.75\mathrm{mdps}/\mathrm{digit}}{0.04\mathrm{dps}/{}^{\circ}C\;\times \left(245\mathrm{dps}/2000\mathrm{dps}\right)+10.9375\mathrm{mdps}/{}^{\circ}C\;}\approx 0.55\;{}^{\circ}C$$

To simplify the test steps, ∆*T* = 0.5 °C. A thermal chamber SET-Z-021 is utilized to test L3GD20H, and its parameters are *C =* 1.005 kJ/(kg×K), *k_h_* = 0.0267 W/m°C, *L* = 0.6 m, and *ρ* = 1.293 kg/m^3^. Given that CMG’s operating temperature range is −40~85 °C, (25) is obtained as follows:

(28) $$t_{s}\approx 25.766\;s$$

So, temperature control units take 25.766 s as the temperature control period to vary the temperature control interval at 0.5 °C. To simplify test steps and reserve an allowance for stable heat conduction, *t_p_* = 30 s. L3GD20H is tested in temperature experiments, and its temperature is obtained with a precise temperature measurement system of measuring accuracy of ±0.03 °C and measuring frequency of 10 Hz. Thus, a temperature experiment is designed as follows:

1. CMG L3GD20H is installed on the rate table, its measuring direction is parallel to the rate table, and the referenced true value is the angular velocity of the rate table.
2. Temperature sensors of the precise temperature measurement system are attached close to L3GD20H, the wireless data transmission module transmits the experimental results, and the PC is prepared to receive its temperature *T_G_* and its output *D_G_*.
3. Cool the thermal chamber to a minimum operating temperature of −40 °C and keep *T_G_* and *D_G_* recording for 0.5 h when the ambient temperature stays stable.
4. Heat the thermal chamber to a maximum operating temperature of 85 °C at a rate of 60 °C/h, 0.5 °C per 30 s. When *T_G_* goes up to 85 °C, stop the test when it stays stable for 0.5 h.
5. Redo steps (2) to (4) five times and record them as the experimental results.

**Figure 6:**
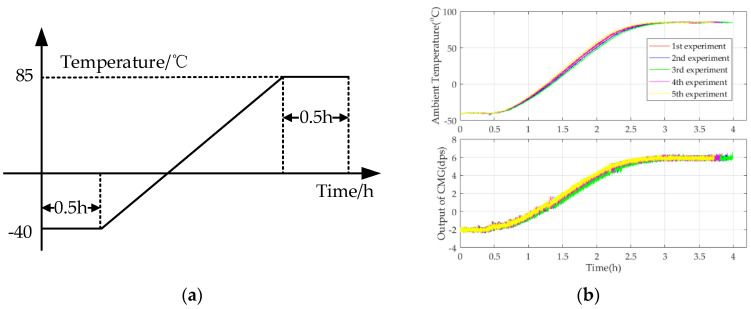
Flowchart of the temperature experiment and experimental results. (**a**) Experimental flow; (**b**) experimental results.

Figure 6 shows a flowchart of the temperature experiment and experimental results.

#### 2.2.2. Implementation of Novel TDE Precise Estimation Model Based on an RBFNN

**Figure 7:**
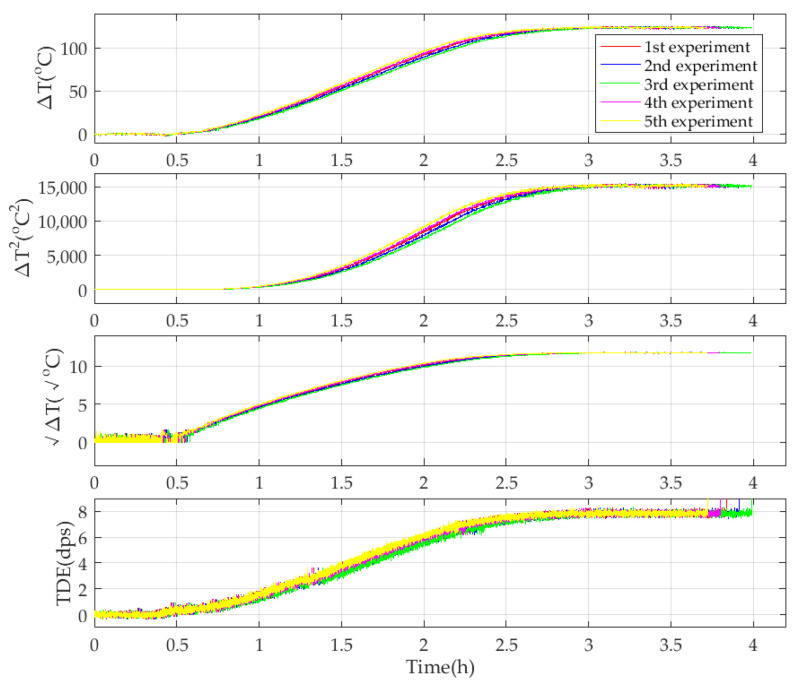
Ambient temperature variation and its square, as well as its square root and TDE.

Under the premise of building a novel TDE precise compensation model and obtaining TDE and TCQs, identifying its precise parameters is another key to estimating the TDE of the CMG accurately. As shown in Figure 6, the ambient temperature goes up from −40 °C to 85 °C, and at the very beginning, the ambient temperature stays stable for 0.5 h, and it is set as the reference ambient temperature. Moreover, the reference output of the CMG is 0 dps/s because $\omega_{s}=0$. ∆*T*, ∆*T*^2^, ∆*T*^1/2^, and TDE deduced from Figure 6 are shown in Figure 7.

**Figure 8:**
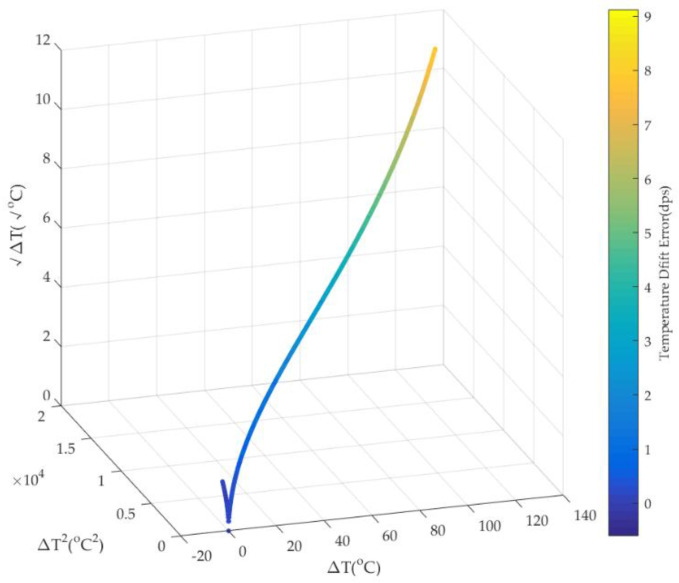
The relationship among ∆*T*, ∆*T*^2^, ∆*T*^1/2^, and TDE.

As shown in Figure 7, when ambient temperature *T* varies, ∆*T* and ∆*T*^2^ vary in a similar trend. Because of the small numerical amplitude of ambient temperature variation at the beginning, ∆*T*^1/2^ has the same trend with a small range of numerical fluctuations. Under the excitation of ∆*T* and ∆*T*^2^, as well as ∆*T*^1/2^, TDE has an approximate varying trend. Therefore, it shows that there is some relevance among ∆*T*, ∆*T*^2^, ∆*T*^1/2^, and TDE. Figure 8 shows the corresponding relationship among them.

As shown in Figure 8, there is a complex nonlinear relationship among ∆*T*, ∆*T*^2^, ∆*T*^1/2^, and TDE, and it is so difficult to estimate precisely in real time. So, it is essential to use a nonlinear model of multiple inputs and a single output as well as high accuracy to fit the nonlinearity. The RBFNN is a feedforward neural network based on the function approximation method with a single hidden layer. It consists of neurons and neural layers. Neurons are applied as basic computing units, and neural layers are utilized as a computing framework. Its neural layers include an input layer, a hidden layer, and an output layer, and neurons are distributed in diverse layers. The neurons in the hidden layer and output layer have kernel functions, and inputs in the RBFNN are transmitted between the input layer and the output layer by neurons and kernel functions. By means of neurons and neural layers, as well as kernel functions, the output in the RBFNN perfectly represents or approximately approaches the targets. Usually, it uses the Gaussian function as the kernel function in the hidden layer and the purelin function as the activation function in the output layer, and they are shown as follows:

(29) $$y=e^{-{\left\|x-c_{j}\right\|}^{2}/2\sigma_{j}^{2}}\;\;\;\;y=purelin\left(x\right)=ax+b$$

where *c_j_* is the center of the Gaussian function and *δ_j_* is its width, *a* and *b* are constant, *x* is the input of the functions, and *y* are their outputs. Moreover, the RBFNN has two merits as follows:

- Owing to RBFNN’s mathematical principles, its calculation results are optimal in global scope to avoid local minimums, even in flat areas where the error gradient approximates to zero.
- From the Kolmogorov theorem, a three-layer forward network is able to approach any continuous function with any target accuracy [18]. The RBFNN has the structure of an input layer, a hidden layer, and an output layer, and it can represent the targeted nonlinearity with any accuracy.

**Figure 9:**
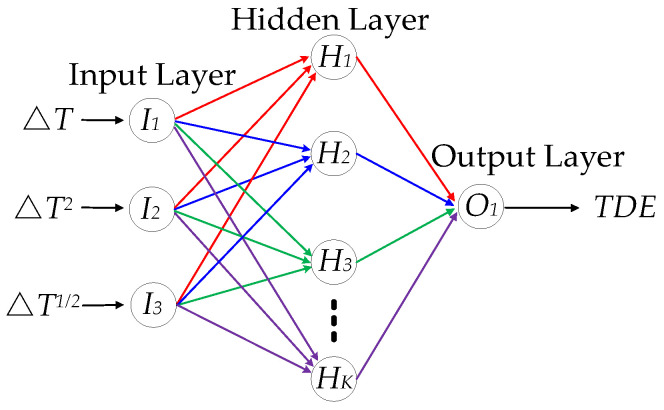
The structure of the RBFNN for the complex nonlinear relationship.

Figure 9 shows the structure of the RBFNN for the complex nonlinear relationship among ∆*T*, ∆*T*^2^, ∆*T*^1/2^, and TDE.

Where *I_i_*(*i* = 1⋯3) is the *i*th neuron of the input layer, *H_i_*(*i* = 1⋯*K*) is *the i*th neuron of the hidden layer, and *O*_1_ is the neuron of the output layer. Based on that, the RBFNN is a good choice to represent the nonlinear relationship among ∆*T*, ∆*T*^2^, ∆*T*^1/2^, and TDE accurately. So, (12) can also be deduced as follows:

(30) $$\Delta E_{cap}\prime =RBFNN\left(\Delta T,\Delta T^{2},\Delta T^{1/2}\right)$$

Then, the parameters of the novel model should be identified as follows:

1. Two temperature experiments are conducted, and the experimental results are recorded. One of them is a training sample set, and the other one is a verification sample set.
2. Based on sample data of the training sample set, TDE is calculated by subtracting the reference value of CMGs from the sample data of their actual outputs one by one. ∆*T* is calculated by subtracting the reference temperature of CMGs from the sample data of their actual temperature one by one. Then, ∆*T*^2^ is obtained by multiplying itself, and ∆*T*^1/2^ is obtained by calculating the square root of ∆*T*.
3. The RBFNN uses ∆*T*, ∆*T*^2^, and ∆*T*^1/2^ as its inputs and TDE as its output. It is trained with mathematical tools until the differences between its outputs and targeted TDE meet the requirements.
4. The compensated results of CMGs are calculated from subtraction between the actual outputs of CMGs and their corresponding estimated outputs of RBFNNs.

**Figure 10:**
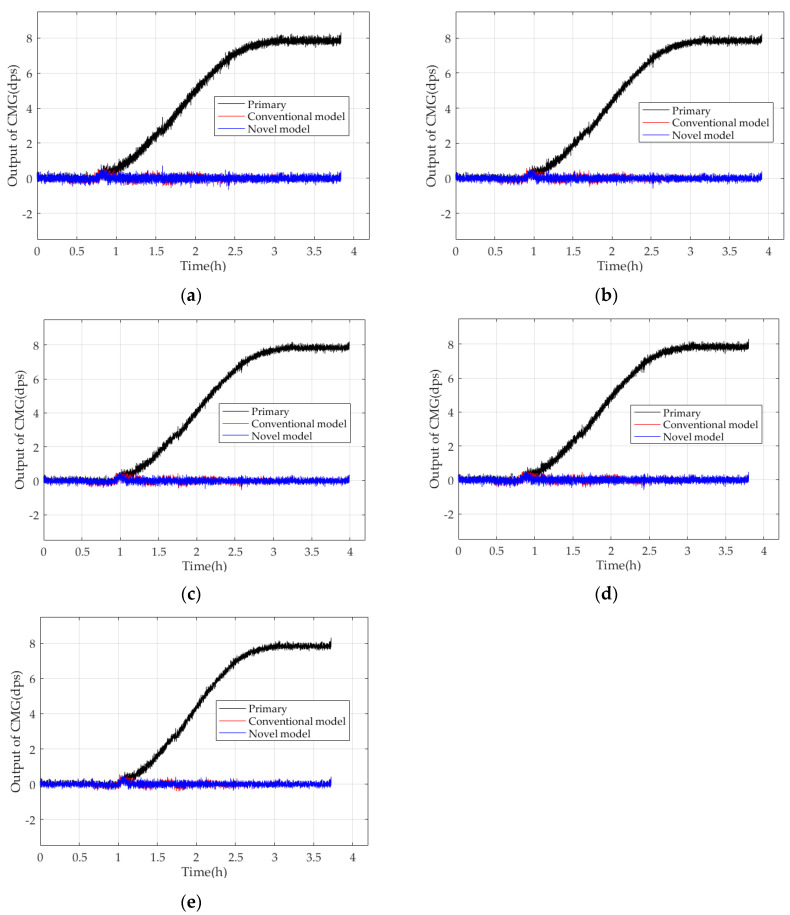
Comparison of the primary data of CMGs and their compensated outputs in five experiments. (**a**) First experiment; (**b**) second experiment; (**c**) third experiment; (**d**) fourth experiment; (**e**) fifth experiment.

The sample data of the training sample set are determined by MEMS Gyros’ output frequency under the target operating conditions. The higher MEMS Gyros’ output frequency is, the more quickly MEMS Gyros outputs, and the higher the sample data of the training sample set. Then, (30) is trained with the experimental results, and its parameters are identified precisely. Figure 10 shows the primary data and those compensated by the conventional model and novel model in five experiments.

To check its estimation performance, the Mean Square Deviation (*MSD*) is introduced as follows:

(31) $$MSD=MSE(x-x^{\prime })$$

where *x* is the evaluated sample, *x*′ is its reference, and the *MSE* is the mean square error algorithm. The *MSD* is an index that indicates the dispersion degree of the evaluated sample and its reference. The less the *MSD* is, the smaller the dispersion degree stays. The bias stability of CMGs is the evaluated sample. So, the *MSD* can be implemented with programming software based on mathematical principles. The *MSD* of the bias stability of its primary data is *MSD*_1_, the bias stability of those compensated by the conventional model is *MSD*_2_, and the bias stability of those compensated by the novel model is *MSD*_3_, which are shown in Table 1. An *MSD* improvement of the conventional model *Q*_1_ and the novel model *Q*_2_ are shown as follows:

(32) $$\begin{array}{cc} Q_{1}=\frac{MSD_{2}}{MSD_{1}} & Q_{2}=\frac{MSD_{3}}{MSD_{1}} \end{array}$$

In Figure 10, CMGs compensated by the conventional and novel models perform stably with small fluctuations around the reference, but CMGs compensated by the novel model run more stably and have smaller fluctuations, which shows that the novel model can estimate TDE more accurately. In Table 1, although the conventional and novel models reduce the *MSD* of the CMG effectively, the novel model reduces the *MSD* of the CMG less, and the *MSD* after compensation is enhanced by four orders of magnitude. Moreover, CMG’s bias stability is enhanced better by 10.5% evenly than the conventional model. So, the novel model can estimate TDE more accurately to improve the bias stability of the CMG significantly.

## 3. Experiments and Analysis

**Figure 11:**
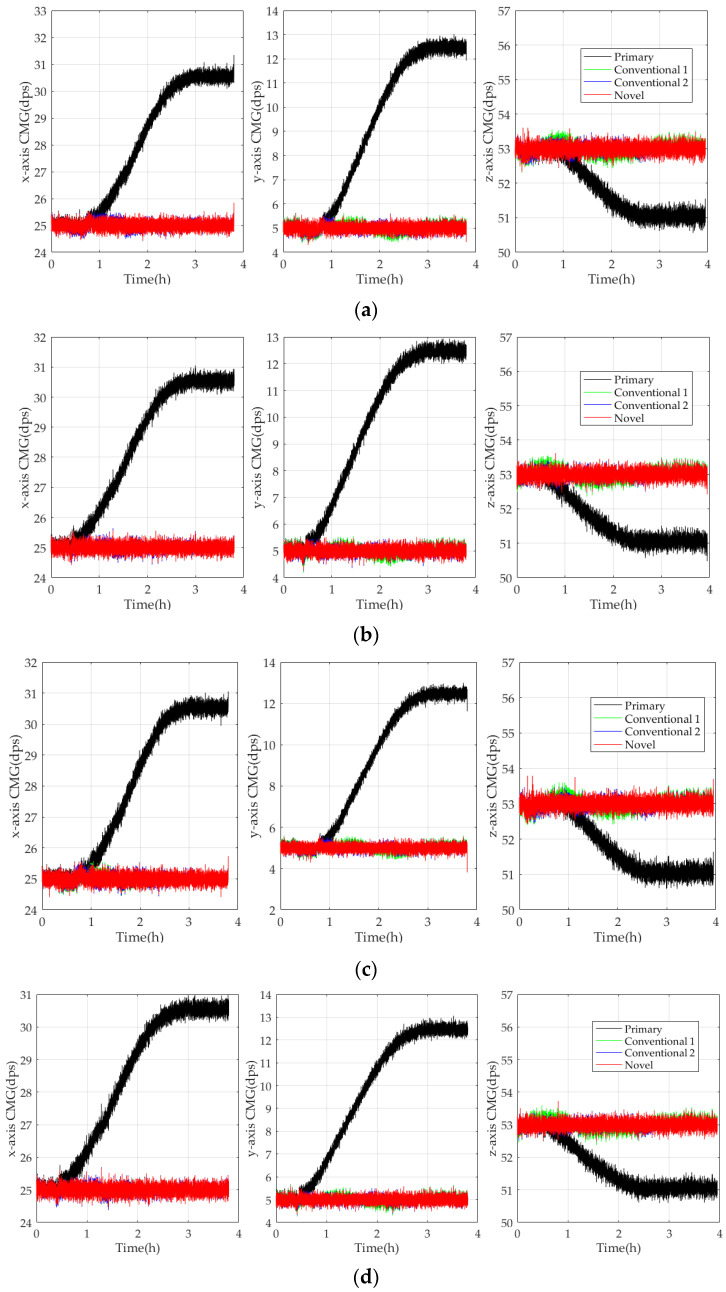

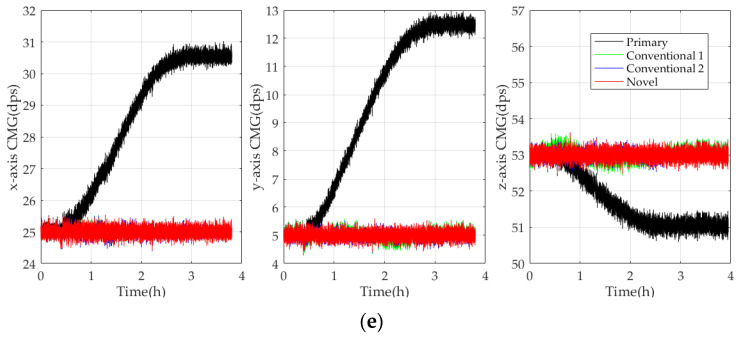
Comparison of the experimental results in five verification experiments. (**a**) First experiment; (**b**) second experiment; (**c**) third experiment; (**d**) fourth experiment; (**e**) fifth experiment.

To verify the estimation universality and the robustness of the novel model further, CMG I3G4250D is taken as a test object. It is chosen randomly and tested in five verification experiments. The conventional model is based on the least square method and ∆*T* (Conventional 1), the conventional one is based on the BPNN and ∆*T* and ∆*T*^2^ (Conventional 2), and the novel one is based on the RBFNN and ∆*T*, ∆*T*^2^, and ∆*T*^1/2^ (Novel). They are established on the *x*-axis, *y*-axis, and *z*-axis, and their bias stabilities are compared. To ensure experimental results’ reliability, the referenced angular velocity in three axes is set as *ω_ref_^x^* = 25 dps, *ω_ref_^y^* = 5 dps, and *ω_ref_^z^* = 53 dps randomly. Figure 11 compares the experimental results in the experiments.

According to (31), the *MSD* of the bias stability of its primary data is *MSD*_1_, the bias stability of those compensated by Conventional 1 is *MSD*_4_, the bias stability of those compensated by Conventional 2 is *MSD*_5_, and the bias stability of those compensated by Novel is *MSD*_6_. They are shown as follows:

(33) $$\begin{array}{l} MSD_{1}=MSE(k-{k^{\prime }}_{i})\\ MSD_{4}=MSE\left[k\left(\Delta T\right)-{k^{\prime }}_{i}\right]\\ MSD_{5}=MSE\left[k\left(\Delta T,\Delta T^{2}\right)-{k^{\prime }}_{i}\right]\\ MSD_{6}=MSE\left[k\left(\Delta T,\Delta T^{2},\Delta T^{-1/2}\right)-{k^{\prime }}_{i}\right] \end{array}$$

where *k* is the primary data of I3G4250D in three axes; *k*(∆*T*) are those compensated by Conventional 1 in three axes; *k*(∆*T*, ∆*T*^2^) are those compensated by Conventional 2 in three axes; *k*(∆*T*, ∆*T*^2^, ∆*T*^1/2^) are those compensated by Novel in three axes; and *k_i_*′(*i* = *x*, *y*, *z*) is the referenced angular velocity in three axes. In (31), the *MSD* improvement of Conventional 1 is *Q*_3_, the *MSD* improvement of Conventional 2 is *Q*_4_, and the *MSD* improvement of Novel is *Q*_5_. *MSD*s in five experiments are shown in Table 2, Table 3, Table 4, Table 5 and Table 6.

(34) $$\begin{array}{ccc} Q_{3}=\frac{MSD_{4}}{MSD_{1}} & Q_{4}=\frac{MSD_{5}}{MSD_{1}} & Q_{5}=\frac{MSD_{6}}{MSD_{1}} \end{array}$$

According to Figure 11, CMGs compensated by Conventional 1, Conventional 2, and Novel perform stably with small fluctuations around the references, and the bias stability of CMGs compensated by the novel model is reduced the most significantly. It shows that the novel model estimates TDE more accurately and effectively, which ensures CMGs perform more stably and reliably. According to Table 2, Table 3, Table 4, Table 5 and Table 6, the *MSD*s of CMGs compensated by Novel are smaller than Conventional 1 and Conventional 2, and its *MSD* after compensation is improved about two orders of magnitude at minimum. Although their difference is small, there is a performance improvement of the test data in a statistical sense. So, the primary data’s performance must be improved at a certain moment; it may be much better or a little better, and its actual performance must be improved more than the *MSD*. Furthermore, Novel and Conventional 2 can enhance CMG’s bias stability better by 15% than Conventional 1, and Novel can enhance CMG’s bias stability more perfectly by 5% than Conventional 2. Hence, the novel model is able to estimate TDE more accurately and enhance the bias stability of CMGs markedly. By comparison, the conventional model has higher TDE estimation accuracy and more perfect universality and robustness, which guarantees CMGs to perform stably and accurately for a long time in diverse complicated conditions.

## 4. Conclusions

In this paper, TDE traceability for CMGs is explored adequately with thermal stress deformation analysis by simulating structural deformation in diverse conditions, and ∆*T,* ∆*T*^2^, and ∆*T*^1/2^ are deduced as more key decisive TCQs to establish the novel TDE precise estimation model. To increase TDE estimation accuracy, an RBFNN is applied to implement the novel model and avoid the local optimums of BPNNs. By analyzing heat conduction between CMGs and the thermal chamber, a precise TDE test is established with heat flux analysis. Two key parameters, the proper temperature control interval and the reasonable temperature control period, are obtained to avoid time-consuming and expensive experiments. The *MSD* is used to evaluate TDE estimation accuracy, and the bias stability of CMGs compensated by the novel model is improved by about 15% compared with the conventional ones. The novel model can estimate TDE more precisely to decouple the temperature dependence of Si-based materials, which enhances the environmental adaptability of CMGs to widen their application in different complicated conditions. This study provides a universal and robust means of estimating the TDE of CMGs in diverse applications, optimizing the TDE precise estimation model to perform precisely and in real time in fields, such as industrial production, smart agriculture, infrastructure construction, and so on, offering a proper TDE precise test method without the need for time-consuming and expensive experiments.

## Figures and Tables

**Table 1:** Performance comparison between the conventional and novel models in experiments.

	*MSD*_1_	*MSD*_2_	*MSD*_3_	*Q*_1_	*Q*_2_	|*Q*_2_ − *Q*_1_|/*Q*_1_ (%)
First experiment	9.5552	3.5555 × 10^−2^	3.2172 × 10^−2^	3.7210 × 10^−3^	3.3670 × 10^−3^	9.51%
Second experiment	9.5579	3.5957 × 10^−2^	3.1756 × 10^−2^	3.7620 × 10^−3^	3.3224 × 10^−3^	11.68%
Third experiment	9.5567	3.4492 × 10^−2^	3.1616 × 10^−2^	3.6092 × 10^−3^	3.3083 × 10^−3^	8.34%
Fourth experiment	9.5590	3.5734 × 10^−2^	3.1339 × 10^−2^	3.7383 × 10^−3^	3.2784 × 10^−3^	12.30%
Fifth experiment	9.5507	3.5442 × 10^−2^	3.1660 × 10^−2^	3.7109 × 10^−3^	3.3150 × 10^−3^	10.67%

**Table 2:** *MSDs* of the experimental results in the first experiment.

	*MSD*_1_	*MSD*_4_	*MSD*_5_	*MSD*_6_	*Q*_3_	*Q*_4_	*Q*_5_	|*Q*_4_ − *Q*_3_|/*Q*_3_ (%)	|*Q*_5_ − *Q*_3_|/*Q*_3_ (%)	|*Q*_5_ − *Q*_4_|/*Q*_4_ (%)
*x*-axis	4.7794	0.0216	0.0214	0.0193	4.519 × 10^−3^	4.478 × 10^−3^	4.038 × 10^−3^	0.93%	10.65%	9.81%
*y*-axis	8.5768	0.0280	0.0233	0.0214	3.265 × 10^−3^	2.717 × 10^−3^	2.495 × 10^−3^	16.79%	23.57%	8.15%
*z*-axis	0.7258	0.0270	0.0220	0.0210	3.720 × 10^−2^	3.031 × 10^−2^	2.893 × 10^−2^	18.52%	22.22%	4.55%

**Table 3:** *MSDs* of the experimental results in the second experiment.

	*MSD*_1_	*MSD*_4_	*MSD*_5_	*MSD*_6_	*Q*_3_	*Q*_4_	*Q*_5_	|*Q*_4_ − *Q*_3_|/*Q*_3_ (%)	|*Q*_5_ − *Q*_3_|/*Q*_3_ (%)	|*Q*_5_ − *Q*_4_|/*Q*_4_ (%)
*x*-axis	3.8545	0.0194	0.0192	0.0184	5.033 × 10^−3^	4.981 × 10^−3^	4.774 × 10^−3^	1.03%	5.15%	4.17%
*y*-axis	6.8265	0.0256	0.0219	0.0208	3.750 × 10^−3^	3.208 × 10^−3^	3.047 × 10^−3^	14.53%	18.75%	5.02%
*z*-axis	0.6582	0.0271	0.0211	0.0202	4.117 × 10^−2^	3.206 × 10^−2^	3.069 × 10^−2^	22.14%	25.46%	4.27%

**Table 4:** *MSDs* of the experimental results in the third experiment.

	*MSD*_1_	*MSD*_4_	*MSD*_5_	*MSD*_6_	*Q*_3_	*Q*_4_	*Q*_5_	|*Q*_4_ − *Q*_3_|/*Q*_3_ (%)	|*Q*_5_ − *Q*_3_|/*Q*_3_ (%)	|*Q*_5_ − *Q*_4_|/*Q*_4_ (%)
*x*-axis	4.7779	0.0210	0.0202	0.0190	4.395 × 10^−3^	4.227 × 10^−3^	3.976 × 10^−3^	3.81%	9.52%	5.94%
*y*-axis	8.5962	0.0289	0.0240	0.0221	3.362 × 10^−3^	2.792 × 10^−3^	2.571 × 10^−3^	16.96%	23.53%	7.92%
*z*-axis	0.7286	0.0280	0.0227	0.0214	3.843 × 10^−2^	3.116 × 10^−2^	2.937 × 10^−2^	24.91%	26.35%	1.92%

**Table 5:** *MSDs* of the experimental results in the fourth experiment.

	*MSD*_1_	*MSD*_4_	*MSD*_5_	*MSD*_6_	*Q*_3_	*Q*_4_	*Q*_5_	|*Q*_4_ − *Q*_3_|/*Q*_3_ (%)	|*Q*_5_ − *Q*_3_|/*Q*_3_ (%)	|*Q*_5_ − *Q*_4_|/*Q*_4_ (%)
*x*-axis	3.8575	0.0200	0.0199	0.0189	5.185 × 10^−3^	5.159 × 10^−3^	4.900 × 10^−3^	0.51%	5.50%	5.03%
*y*-axis	6.8208	0.0256	0.0221	0.0211	3.753 × 10^−3^	3.240 × 10^−3^	3.094 × 10^−3^	13.67%	17.58%	4.52%
*z*-axis	0.6538	0.0273	0.0207	0.0200	4.176 × 10^−2^	3.166 × 10^−2^	3.059 × 10^−2^	24.18%	26.74%	3.38%

**Table 6:** *MSDs* of the experimental results in the fifth experiment.

	*MSD*_1_	*MSD*_4_	*MSD*_5_	*MSD*_6_	*Q*_3_	*Q*_4_	*Q*_5_	|*Q*_4_ − *Q*_3_|/*Q*_3_ (%)	|*Q*_5_ − *Q*_3_|/*Q*_3_ (%)	|*Q*_5_ − *Q*_4_|/*Q*_4_ (%)
*x*-axis	3.8538	0.0198	0.0193	0.0189	5.138 × 10^−3^	5.008 × 10^−3^	4.904 × 10^−3^	2.53%	4.55%	2.07%
*y*-axis	6.8339	0.0256	0.0217	0.0212	3.746 × 10^−3^	3.175 × 10^−3^	3.102 × 10^−3^	15.23%	17.19%	2.30%
*z*-axis	0.6588	0.0274	0.0209	0.0203	4.159 × 10^−2^	3.172 × 10^−2^	3.081 × 10^−2^	23.72%	25.91%	2.87%

## Data Availability

Data are contained within the article.
